# Comprehensive Analysis of Antiphage Defense Mechanisms: Serovar-Specific Patterns

**DOI:** 10.3390/antibiotics13060522

**Published:** 2024-06-03

**Authors:** Pavlo Petakh, Valentyn Oksenych, Yevheniya Khovpey, Oleksandr Kamyshnyi

**Affiliations:** 1 Department of Biochemistry and Pharmacology, Uzhhorod National University, 88000 Uzhhorod, Ukraine; pavlo.petakh@uzhnu.edu.ua (P.P.); mf.khovpei.yevheniia@student.uzhnu.edu.ua (Y.K.); 2 Department of Microbiology, Virology and Immunology, I. Horbachevsky Ternopil National Medical University, 46001 Ternopil, Ukraine; 3 Broegelmann Research Laboratory, Department of Clinical Science, University of Bergen, 5020 Bergen, Norway

**Keywords:** leptophages, Cas system, bacteriophages, *Leptospira interrogans*

## Abstract

Leptospirosis is a major zoonotic disease caused by pathogenic spirochetes in the genus Leptospira, affecting over a million people annually and causing approximately 60,000 deaths. *Leptospira interrogans*, a key causative agent, likely possesses defense systems against bacteriophages (leptophages), yet these systems are not well understood. We analyzed 402 genomes of *L. interrogans* using the DefenseFinder tool to identify and characterize the antiphage defense systems. We detected 24 unique systems, with CRISPR-Cas (Clustered Regularly Interspaced Short Palindromic Repeats and CRISPR-associated proteins), PrrC, Borvo, and Restriction-Modification (R-M) being the most prevalent. Notably, Cas were identified in all strains, indicating their central role in phage defense. Furthermore, there were variations in the antiphage system distribution across different serovars, suggesting unique evolutionary adaptations. For instance, Retron was found exclusively in the Canicola serovar, while prokaryotic Argonaute proteins (pAgo) were only detected in the Grippotyphosa serovar. These findings significantly enhance our understanding of *Leptospira*’s antiphage defense mechanisms. They reveal the potential for the development of serovar-specific phage-based therapies and underscore the importance of further exploring these defense systems.

## 1. Introduction

Leptospirosis, one of the most common but globally neglected zoonotic infections caused by pathogenic spirochetes of the genus *Leptospira*, significantly impacts global human health, affecting more than a million people and causing approximately 60,000 deaths annually [1,2,3]. Current recommendations for the treatment of human leptospirosis involve beta-lactam antibiotics such as penicillin, ampicillin, ceftriaxone, or cefotaxime [4,5,6]. Alternative therapeutic options for leptospirosis are critical for patients who cannot tolerate beta-lactam antibiotics due to allergies or other contraindications. Among these options, oral doxycycline and azithromycin are frequently used [7,8,9,10].

While antibiotic treatment effectively targets infections, it can also disrupt the body’s natural microbiota, leading to several adverse effects. One significant consequence is gut dysbiosis, which involves a reduction in microbial diversity and shifts in the relative abundance of specific bacteria. This can lead to the overgrowth of harmful taxa, such as *Clostridium difficile*, increasing the risk of pseudomembranous colitis [11,12,13]. *Leptospira* species demonstrate intrinsic resistance to various antimicrobial agents, although the precise mechanisms responsible for this resistance are not yet fully understood [14,15]. Leptospira shows resistance to sulfonamides, neomycin, actidione, polymyxin, nalidixic acid, vancomycin, and rifampicin, which has allowed the creation of selective nutrient media for its isolation [16]. Due to their tendency to target specific hosts, phages generally have a minimal impact on beneficial bacteria and may be good therapeutic options in the future [17].

Leptospira species primarily inhabit damp environments, where they are prone to encountering diverse phages and plasmids, in addition to their ability to infect both humans and animals. In 1990, Saint Girons et al. isolated three bacteriophages from sewage waters near Paris, France that infected the saprophytic bacterium *Leptospira biflexa*. These phages, named LE1 (vB_LbiM_LE1), LE3 (vB_LbiM_LE3), and LE4 (vB_LbiM_LE4), were found to be lytic and had a host range limited to serovar Patoc of *L. biflexa* [18]. Electron micrographs revealed that the phages shared a morphological structure with polyhedral heads and contractile tails. In 2018, Schiettekatte sequenced and analyzed the genomes of the lytic phages LE3 and LE4, which infect *L. biflexa*, using the lipopolysaccharide O-antigen as a receptor. Further investigation revealed a related plasmid in *L. interrogans* and a prophage-like region in the genome of a clinical isolate of *L. mayottensis*, suggesting genetic connections between leptophages and other *Leptospira* species [19,20]. 

Considering the presence of leptophages, it is likely that leptospires have appropriate natural protection systems against phages to limit phage infection [21]. Xiao et al. described the presence of the CRISPR-Cas system; however, as is known, microorganisms have a large arsenal of defense systems against phages [22,23]. Antiphage defense systems exhibit a nonrandom distribution in microbial genomes, often forming “defense islands” where multiple systems cluster together [24,25,26]. Several studies have revealed that different strains of the same bacterial species may encode distinct defense systems [26]. A limitation of these studies is that they primarily focus on specific antiphage defense systems, such as CRISPR-Cas and Restriction-Modification (R-M), while neglecting to comprehensively investigate the entire spectrum of antiphage defense systems present in *Leptospira*. 

The rationale for the investigation of antiphage systems in leptospirosis lies in more than simply filling a research gap. With bacteriophages emerging as a potential therapeutic alternative to antibiotics, understanding how Leptospira strains resist phage attacks becomes critical. By examining the diversity and distribution of the antiphage defense systems in Leptospira, we can identify possible challenges to phage-based therapy and gain insights into the mechanisms that underlie bacterial survival strategies. This line of inquiry offers a more nuanced understanding of Leptospira’s adaptability and informs future efforts to leverage or overcome these defense systems in clinical contexts.

Therefore, this study aimed to comprehensively identify and analyze the antiphage defense systems in *L. interrogans* strains and serovars. This comprehensive approach provides a fuller understanding of the leptospiral antiphage landscape, with implications for the development of new therapeutic strategies and the expansion of scientific knowledge in this field.

## 2. Results

### 2.1. Phylogenetic Analysis

**Figure 1:**
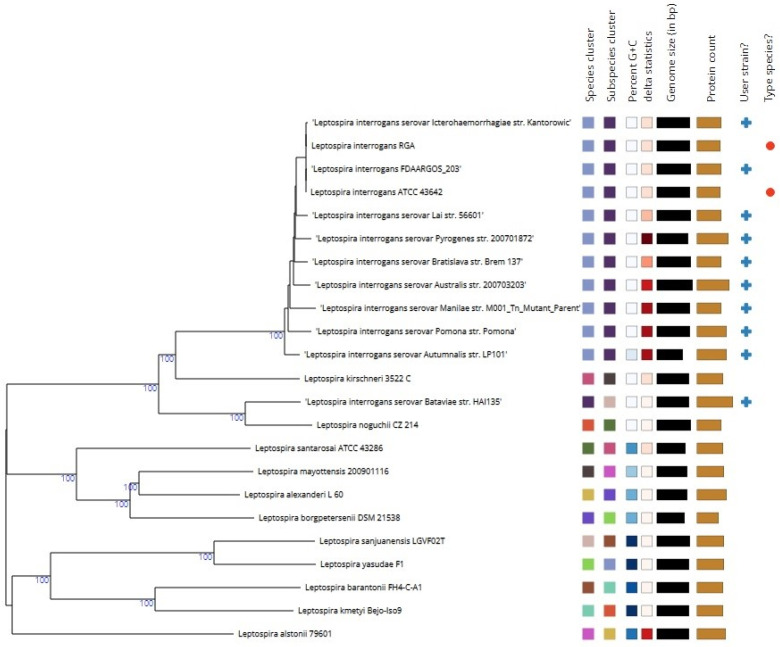
Tree inferred with FastME 2.1.6.1 from GBDP distances calculated from genome sequences. The branch lengths are scaled in terms of GBDP distance formula d5. The numbers above the branches are GBDP pseudo-bootstrap support values > 60% from 100 replications, with average branch support of 62.9%. The tree was rooted at the midpoint.

The analysis identified type-based species and subspecies clusters. Figure 1 illustrates the phylogenetic tree derived from the whole-genome sequences. This tree was constructed using FastME 2.1.6.1, based on the Genome BLAST Distance Phylogeny (GBDP) distances calculated with the d5 distance formula. Pseudo-bootstrap support values above 60%, derived from 100 replications, are displayed above the branches, with average branch support of 62.9%. This genome-based phylogenetic tree provides a comprehensive overview of the evolutionary relationships among the analyzed strains (Figure 1).

### 2.2. General Distribution of Antiphage Systems

In total, 24 antiphage systems were identified among all studied strains. The most widespread systems were Cas (n = 402 strains), PrrC (n = 391 strains), Borvo (n = 388 strains), and R-M (R-M_Type_IV, n = 348 strains; RM_Type_I, n = 27 strains; RM_Type_II, n = 7 strains; RM_Type_IIG, n = 47 strains). We identified the following types of CRISPR-Cas systems: CAS_Class1-Subtype-I-B in 354 strains, CAS_Class1-Subtype-I-C in 399 strains, CAS_Class2-Subtype-V-A in 2 strains, and CAS_Cluster in 149 strains. Moreover, we identified several subtypes—CAS_Class1-Subtype-I-E, CAS_Class1-Subtype-IV-B, and CAS_Class1-Type-I—which were each detected in only one strain.

**Figure 2:**
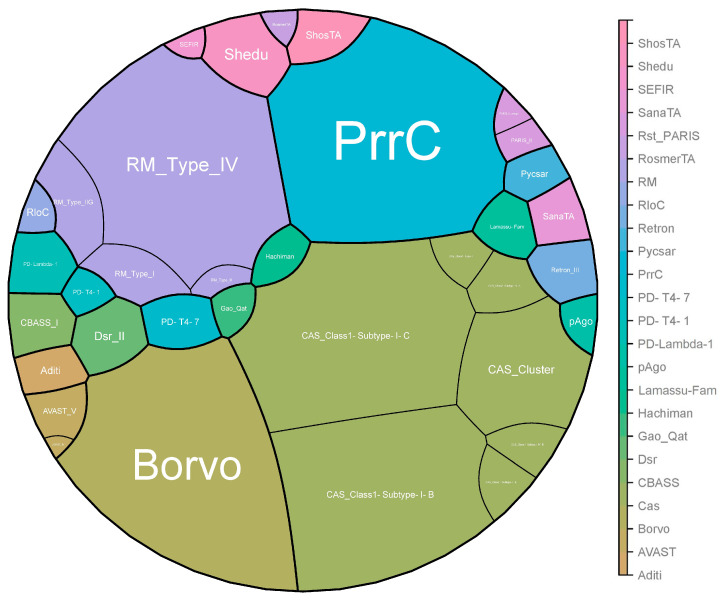
Voronoi treemaps of antiphage defense system distribution. In total, we identified 24 antiphage systems. Four major antiphage systems are present in the vast majority of strains, namely Cas, PrrC, Borvo, and R-M. The image was generated using SrPlot (https://www.bioinformatics.com.cn/, accessed on 12 January 2024). Voronoi treemaps visualize hierarchical data by recursively partitioning convex polygons using weighted centroidal Voronoi diagrams. The polygon areas are proportional to the relative weights of their corresponding nodes. The size of each polygon indicates the frequency of the corresponding antiphage system. The clustering within the treemap represents the hierarchical relationships among different antiphage systems, and the color scale is used to differentiate between various systems and their prevalence.

The rarest systems were AVAST_IV, Gao_Qat, PD-T4-1, and RosmerTA, each of which was detected in only one strain (Figure 2).

### 2.3. Serovar-Specific Findings

**Figure 3:**
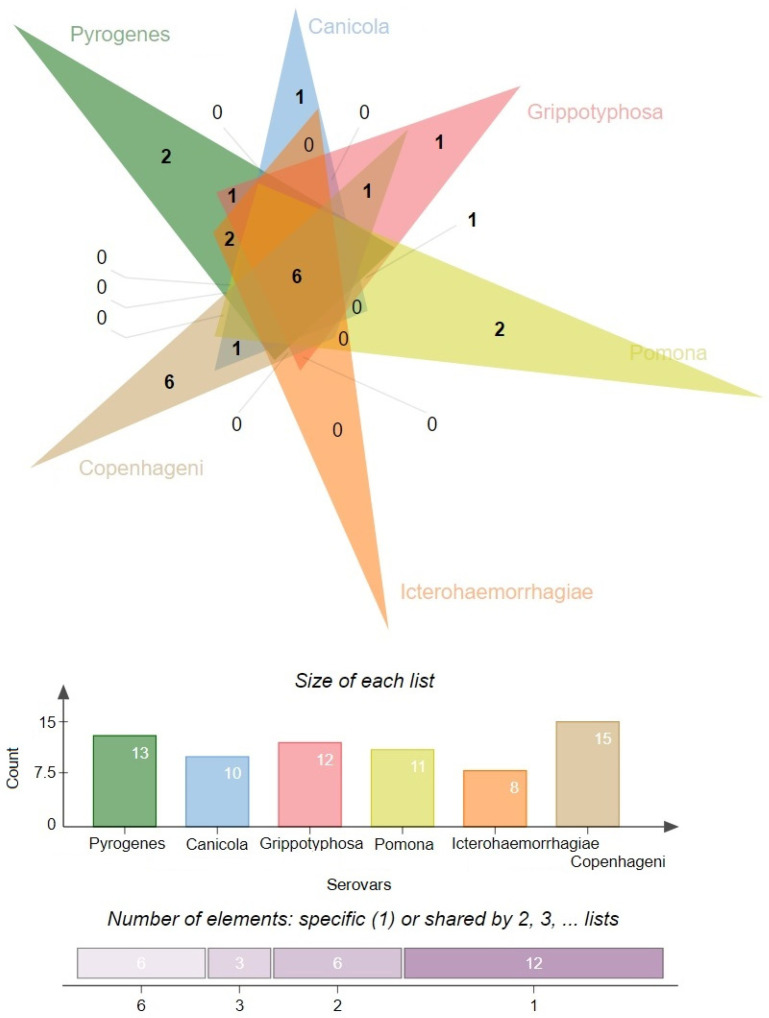
Distribution of defense systems across 6 main serovars (Venn diagram). The most diverse system was found for serovar Copenhageni (n = 15) and serovar Pyrogenes (n = 13), while the least diverse system was found for serovar Icterohemorrhagiae (n = 8). The images were generated using jvenn [27].

We also analyzed the diversity of the antiphage systems depending on the serovar (Figure 3). The following six main serovars were considered: Pyrogenes, Canicola, Grippotyphosa, Pomona, Icterohaemorrhagiae, and Copenhageni. Among all of the antiphage systems that have been identified, the Cas system is present in all serovars. This shows that it is widely used as a defense mechanism. Similarly, the PrrC system has high prevalence, being present at 90% or more in all serovars except Pyrogenes, where it is observed at a 100% frequency.

The R-M system, on the other hand, exhibits variable prevalence across different serovars, ranging from 86.67% in Pomona to 100% in Canicola and Icterohaemorrhagiae. The Borvo system is found at a frequency of 75% in Grippotyphosa and 100% in other serovars (Canicola, Icterohaemorrhagiae).

**Figure 4:**
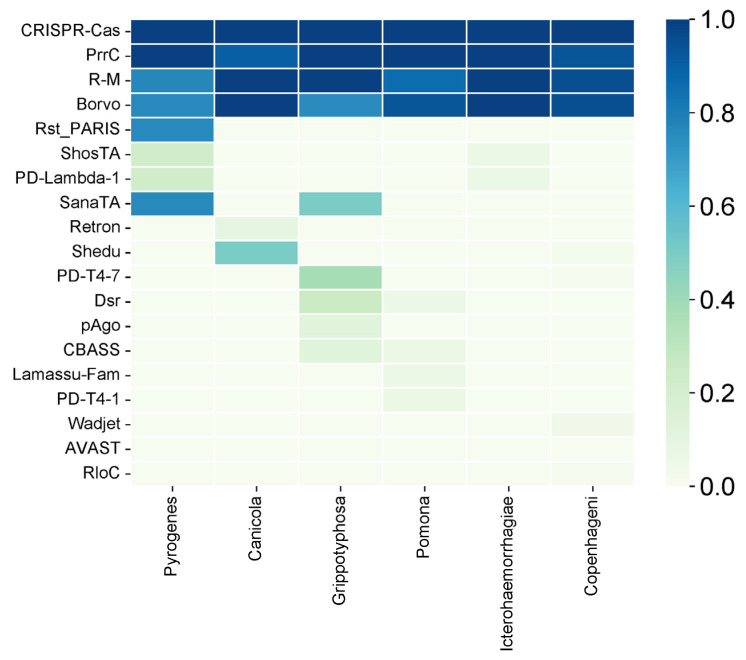
Heatmap of the distribution of defense systems across 6 main serovars. The Cas system stands out for its uniform presence across all serovars, indicating its widespread occurrence as a defense mechanism with a 100% (1.0) frequency. Conversely, certain antiphage systems, such as Retron, Shedu, PD-T4-7, Dsr, pAgo, CBASS, Lamassu-Fam, PD-T4-1, Wadjet, AVAST, and RloC, demonstrate lower frequencies and are occasionally exclusive to specific serovars. The images were generated using SrPlot.

Several less common antiphage systems, including ShosTA, PD-Lambda-1, Rst_PARIS, and SanaTA, exhibit varying degrees of occurrence among the serovars. ShosTA, for instance, is detected at a 23.08% frequency in Pyrogenes and a 6.25% frequency in Icterohaemorrhagiae. PD-Lambda-1 is detected at 23.08% in Pyrogenes and 6.25% in Icterohaemorrhagiae. Rst_PARIS is present at 7.69% in Pyrogenes. The frequency of SanaTA is 50% in Grippotyphosa (Figure 4).

Some antiphage systems, such as Retron, Shedu, PD-T4-7, Dsr, pAgo, CBASS, Lamassu-Fam, PD-T4-1, Wadjet, AVAST, and RloC, show lower frequencies and are sometimes only found in certain serovars. For example, Shedu is detected in 50% of the Canicola serovar and 2.04% of the Copenhageni serovar. PD-T4-7 is found in 37.5% of the Grippotyphosa serovar and 1.02% of the Copenhageni serovar. Dsr is present in 25% of the Grippotyphosa serovar and 6.67% of the Pomona serovar. pAgo is detected in 12.5% of the Grippotyphosa serovar. CBASS is found in 12.5% of the Grippotyphosa serovar and 6.67% of the Pomona serovar. Lamassu-Fam is present in 6.67% of the Pomona serovar. PD-T4-1 is detected in 6.67% of the Pomona serovar. Wadjet is detected in 4.08% of the Copenhageni serovar, and RloC is detected in 1.02% of the Copenhageni serovar.

## 3. Discussion

**Figure 5:**
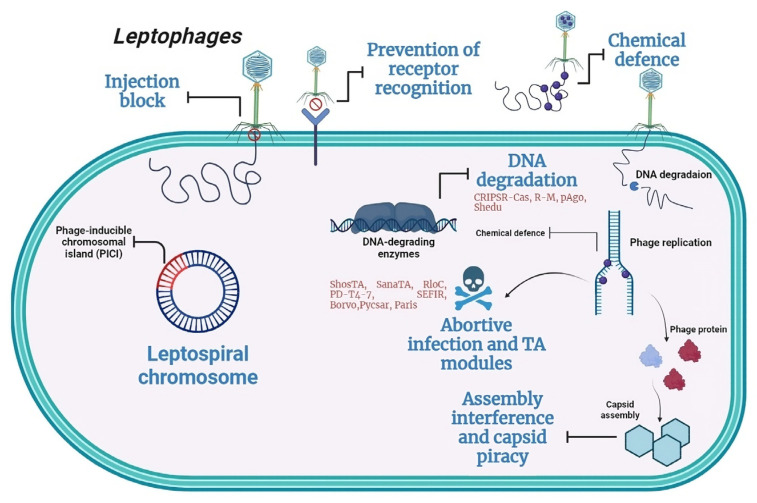
General outline of leptospiral defense strategies targeting different stages of the leptophage cycle. Leptospira strains possess various anti-leptophage systems, such as CRISPR-Cas, R-M, pAgo, and Shedu, which affect DNA degradation. Additionally, they exhibit antiphage systems that lead to abortive infection, as exemplified by Borvo and Shosta. The images were generated using BioRender (https://www.biorender.com, accessed on 12 January 2024).

A recent pangenome analysis has unveiled various new systems within bacterial genomes, some of which elucidate novel defense mechanisms at the population level [24]. These bacterial defense systems pose challenges to the effectiveness of phage application. The thorough characterization of phage defense systems, along with their potential drawbacks, is essential for the deeper comprehension of the hurdles in practical phage application and for the advancement of phage technology. Of the 151 antiphage systems known today, 24 were found in the investigated *Leptospira* strains (Figure 5) [28].

Just as antibiotic resistance genes confer bacterial resistance, genes from defense systems can equip host cells with the ability to fend off phages. These defense mechanisms are commonly observed in bacteria. For instance, the Restriction-Modification (RM) system, initially discovered in the 1950s, is highly prevalent and is present in approximately 90% of diverse bacterial strains [29].

The functional subunits of R-M systems include methyltransferase (MTase), which transfers a methyl group from the S-adenosyl methionine (SAM) donor molecule to cytosine or adenine in the DNA, along with the corresponding restriction endonuclease (REase). Certain systems additionally encode a translocase that employs ATP hydrolysis energy for motor functions. They also feature a specificity subunit containing target recognition domains (TRDs), which define the sequence specificity of the REase and MTase. R-M systems are categorized into four types based on their subunit composition, co-factor requirements, and mode of action [30]. Type II R-M systems typically recognize palindromic sites, cleaving both DNA strands within or near the nonmethylated sites [31]. Type I R-M systems modify both strands of bipartite asymmetric DNA sites, requiring an interaction between two restriction complexes bound to nonmethylated sites via ATP-dependent DNA looping. Cleavage occurs at nonfixed positions between sites. Type III R-M systems modify only one strand of asymmetric recognition sites, cleaving at a fixed position from one recognition site. This occurs when the restriction complex bound to the nonmethylated site interacts with another complex activated by the recognition of a nearby nonmethylated site in the inverted repeat orientation. Type IV R-M systems lack a modification module and cleave DNA after recognizing modified sites. Among the Leptospira strains studied, type IV R-M (n = 348), type I R-M (n = 27), and type II R-M (n = 7) were the most common. Type III R-M was not detected in any of the studied strains.

Unlike DNA modification-based innate immune systems, which rely on predetermined sequence interactions with defense proteins within a phage genome, prokaryotes possess adaptive immune CRISPR-Cas systems. Introduced decades after R-M, CRISPR-Cas comprise a CRISPR array and associated Cas genes. A CRISPR array consists of short repeated DNA fragments separated by unique spacer sequences, some originating from foreign DNA, with an AT-rich leader region preceding it. Classification is based on the effector complex protein composition, yielding two classes, six types, and 33 subtypes. The class 1 (types I, III, and IV) systems use multi-subunit effectors, while the class 2 (types II, V, and VI) systems employ single-subunit effectors [32]. CRISPR-Cas systems are present in less than half of all bacteria and in most Archaea [33]. Type I Cas systems are the most prevalent and are found in 30% of all genomes, followed by type II (8%) and type III (6%) systems, as demonstrated in research by Aude Bernheim et al. [34]. All three genes were distributed across multiple phyla. Types IV, V, and VI are exceedingly rare; have been identified in fewer than 70 genomes; and are primarily confined to specific clades, such as Proteobacteria, Actinobacteria, and Bacteroidetes. Only two studies on Leptospira antiphage systems can be identified, with both studies exclusively focused on CRISPRs and their subtypes [22,35]. The dominant CRISPR types for Leptospira are CRISPR types I (the majority of pathogenic species harbor two different types of CRISPR-Cas systems (subtype I-B and subtype I-E)) and III. In our study, we identified the following types and subtypes of Cas systems: CAS_Class1-Subtype-I-C (n = 399 strains), CAS_Class1-Subtype-I-B (n = 354 strains), and CAS_Cluster (n = 149 strains). Rare types were found, including CAS_Class2-Subtype-V-A in two strains (*Leptospira interrogans* serovar Copenhageni str. Fiocruz LV4231 and *Leptospira interrogans* serovar Copenhageni str. Fiocruz LV3992), CAS_Class1-Subtype-IV-B in one strain (*Leptospira interrogans* str. HAI1536), and CAS_Class1-Subtype-I-E in one strain (*Leptospira interrogans* serovar Bataviae str. HAI135). According to the relevant data, the CRISPR-Cas system was identified in more than 37% of prokaryotic strains, with the same frequency across all prokaryotic phyla except Chlamydiota and Mycoplasmatota.

The third most common antiphage system across the *L. interrogans* strains was PrrC, an anticodon nuclease that specifically targets tRNALys. This system supports the R-M system by breaking down tRNALys and suppressing phage proliferation [36,37,38]. PrrC is categorized as an abortive infection system due to its disruption of the host translation machinery [36,37,38,39]. According to the literature, among the 22,803 complete genomes in RefSeq, PrrC is detected in 705 genomes (3.09%), with a much higher distribution in the Verrucomicrobiota phylum (40%) [40].

The last of the most frequently encountered defense systems is Borvo, which is a single-gene antiphage system acquired through a combination of bioinformatic prediction and experimental validation [41]. Borvo is potentially an abortive infection mechanism, but, to the best of our knowledge, the precise molecular mechanism underlying Borvo activity remains unidentified [41,42]. Regarding the prevalence of this antiphage system, the Borvo gene was found in 177 genomes, accounting for 0.78% of the total. It was more frequent in the phyla Bacteroidota and Pseudomonadota, each with a frequency of 1.2% [43]. Although this antiphage system is relatively rare, we were able to detect it in most of our strains.

The Shedu antiphage system was used for half of the Canicola strains. It consists of a single protein, SduA, which acts as a nuclease with a conserved DUF4263 domain belonging to the PD-(D/E)XK nuclease superfamily. The Shedu protein is proposed to act as a nuclease, and its N-terminal domain inhibits its activation until it is triggered by phage infection [44]. Its frequency is relatively low, accounting for only 3.84% in other bacteria, with higher prevalence in the phyla Cyanobacteria and Thermodesulfobacteriota (around 7%) [24].

PD-T4-7, found in 37.5% of Grippotyphosa strains, is a single-gene system that functions through an abortive infection mechanism. Among the 22,803 complete RefSeq genomes, PD-T4-7 was detected in 155 (0.68%) [45]. Dsr, which is also present in 25% of Grippotyphosa strains, has two subsystems. In our strains, we found only subsystem II. Among the more than twenty-two thousand complete RefSeq genomes, Dsr was detected in 246 genomes across 162 different species (1.08%), with higher prevalence in the phyla Thermodesulfobacteriota, Pseudomonadota, and Spirochaetota.

## 4. Materials and Methods

### 4.1. Antiphage System Analysis

We downloaded 402 genomes of *Leptospira interrogans* in FASTA format from the Bacterial and Viral Bioinformatics Resource Center (BV-BRC) database in December 2023 (consisting of 52 complete genomes and 350 whole-genome shotgun (WGS) sequences) [46]. The rationale for the selection of these 402 genome sequences was that we aimed to use all complete and whole-genome shotgun (WGS) sequences of *Leptospira interrogans* available on the Bacterial and Viral Bioinformatics Resource Center (BV-BRC) platform. This comprehensive approach ensured that our analysis covered the full genetic diversity of *L. interrogans*, allowing us to accurately identify and compare the presence of antiphage defense systems across a wide range of strains and serovars.

To identify antiphage defense systems, we employed DefenseFinder (https://defensefinder.mdmlab.fr, accessed on 20 December 2023), a specialized computational tool designed to detect antiviral defense systems in prokaryotic genomes [43,47]. DefenseFinder is built on MacSyFinder, which uses a two-step approach to identifying relevant sequences. Initially, proteins are detected using hidden Markov model (HMM) profiles. Subsequently, decision rules are applied to filter hits based on their genetic architecture, allowing us to identify and classify various defense systems accurately [48]. This method was chosen for its proven reliability in detecting a diverse range of antiphage systems, providing a comprehensive view of the defense landscape in each genome.

After identifying potential defense systems with DefenseFinder, we conducted statistical analyses and data visualization to evaluate the distribution and prevalence of these systems across the 402 genomes. Tools such as SRplot, jvenn, and Python (https://colab.research.google.com/ accessed on 29 December 2023) were used to generate visual representations of the data, aiding in the analysis of overlap and unique occurrences among the various defense systems. These visualizations provided a clearer understanding of the relative frequency of specific antiphage systems within different strains and serovars [27,49].

### 4.2. Phylogenetic Analysis

We performed a whole-genome-based taxonomic analysis using the Type (Strain) Genome Server (TYGS) [50]. This platform allows for comprehensive genomic comparisons and is continually updated with new methodological features [51]. In addition to the genomic analysis, the List of Prokaryotic Names with Standing in Nomenclature (LPSN) database was used for the nomenclature and taxonomic literature [51]. The TYGS analysis was subdivided into the following steps.

#### 4.2.1. Determination of Closely Related Type Strains

To identify closely related type strains, we employed two complementary approaches. First, all user genomes were compared against all available type strain genomes in the TYGS database using the MASH algorithm, selecting the ten type strains with the smallest MASH distances for each user genome [52]. Second, the 16S rDNA gene sequences were extracted from the user genomes using RNAmmer [4], and each sequence was BLASTed [5] against the 16S rDNA gene sequences of the 20,957 type strains in the TYGS database [53,54]. From the BLAST results, the best 50 matches (according to the bitscore) were used to calculate precise distances using the Genome BLAST Distance Phylogeny (GBDP) approach, under the “coverage” algorithm and distance formula d5. This information was used to determine the 10 closest type strain genomes for each user genome.

#### 4.2.2. Pairwise Comparison of Genome Sequences

For the phylogenomic inference, we conducted all pairwise comparisons among the set of genomes using GBDP with accurate intergenomic distances under the “trimming” algorithm and distance formula d5 [55]. We calculated 100 distance replicates for each comparison. Digital DDH values and confidence intervals were calculated using the recommended settings of the GGDC 4.0 [55].

#### 4.2.3. Phylogenetic Inference

Using the intergenomic distances, we inferred a balanced minimum evolution tree with branch support via FASTME 2.1.6.1, including SPR post-processing [56]. Branch support was determined from 100 pseudo-bootstrap replicates, and the trees were rooted at the midpoint. The visualizations were created using PhyD3 [57]. 

The expected outcome of this study was a detailed map of the antiphage defense systems in Leptospira interrogans, highlighting common and unique systems and illustrating their distribution patterns. By identifying these systems across a broad dataset, we aimed to contribute to the knowledge base on Leptospira’s inherent resistance mechanisms and provide insights into potential barriers to phage therapy. The raw data used for this analysis are available in the Supplementary Materials for further reference and validation.

## 5. Limitations

This study characterized the antiphage defense systems in *Leptospira interrogans* using genomic analysis, but it had some limitations. First, we relied on computational tools to detect the defense systems, which might have missed systems with atypical genetic structures or those not represented in the databases used to train these algorithms. Second, our focus on genomics did not account for regulatory mechanisms or post-transcriptional modifications that could affect the expression and activity of antiphage systems. Third, the study did not examine how these defense systems interact with known leptophages, leaving gaps in our understanding of their functional relevance.

## 6. Conclusions

We identified four major antiphage systems present in the vast majority of strains, namely Cas, PrrC, Borvo, and R-M. The significant prevalence of the relatively uncommon Borvo antiphage system in the studied strains underscores the importance of further research in this field. Additionally, certain serovar-dependent features were revealed. Retron was found only in serovar Canicola, pAgo was found only in serovar Grippotyphosa, and Wadjet and RloC were found only in serovar Copenhageni.

Our results provide a foundational understanding of the antiphage defense landscape in *L. interrogans*, which could have implications for the development of phage-based therapies for leptospirosis. Future research should aim to experimentally validate these systems, investigate their interactions with leptophages, and explore their role in resistance mechanisms. This deeper understanding could guide the development of more effective therapeutic strategies and reveal new insights into bacterial defense mechanisms.

## Data Availability

The original contributions presented in the study are included in the article/Supplementary Materials, and further inquiries can be directed to the corresponding authors.

## Supplementary Materials

The following supporting information can be downloaded at: https://www.mdpi.com/article/10.3390/antibiotics13060522/s1.
